# Maintenance of Postoperative Reduction in Trochanteric Fracture Fixation: A Prospective Comparison of Helical Blades and Screws in a Single Cephalomedullary Nailing System

**DOI:** 10.2106/JBJS.OA.25.00073

**Published:** 2025-10-22

**Authors:** Matthias Wittauer, Joseph Henry, Christopher W. Jones, Piers J. Yates

**Affiliations:** 1 Department of Orthopaedic Surgery, Fremantle and Fiona Stanley Hospitals, Perth, Australia; 2 The Orthopaedic Research Foundation of Western Australia (ORFWA), Perth, Australia; 3 Faculty of Medicine, University of Basel, Basel, Switzerland; 4 Division of Surgery, University of Western Australia, Perth, Australia

## Abstract

**Background:**

Cephalomedullary nailing systems, such as the trochanteric femoral nail advanced (TFNa), are standard of care for trochanteric fractures. The TFNa system allows for the use of either a helical blade or a lag screw for femoral neck fixation, but there is ongoing debate regarding which component provides superior outcomes. The aim of this study was to compare the performance of these two components in maintaining fracture reduction and preventing mechanical complications.

**Methods:**

A prospective cohort study enrolled 201 patients aged 50 years or older with type 31A1-31A2 (pertrochanteric) and 31A3 (intertrochanteric) fractures, as classified by the Arbeitsgemeinschaft für Osteosynthesefragen (AO)/Orthopaedic Trauma Association (OTA), all treated with the short TFNa. Patients were allocated to helical blade (n = 101) or lag screw (n = 100) cohorts. Radiographic outcomes—changes in the tip-apex distance (TAD), femoral neck shortening, and changes in the neck-shaft angle (NSA)—were assessed at 3 months. Secondary outcomes included mechanical complications, patient-reported outcome measures (PROMs), and number of patients deceased at 1-year follow-up.

**Results:**

Radiographic outcomes among pertrochanteric fractures—femoral neck shortening, NSA, and TAD changes—were comparable between cohorts. Owing to limited power, no conclusions could be drawn for intertrochanteric fractures. Mechanical complication rates were similar between groups (blades: 4.0%, screws: 6.0%), with no significant association between femoral neck component and complications. A postoperative TAD greater than 20 mm was significantly associated with increased mechanical complications (odds ratio = 4.4, p = 0.023). PROMs improved similarly in both groups over time, and the number of patients deceased within 1 year after the operation was identical in both cohorts.

**Conclusions:**

The findings indicate that helical blades and lag screws offer comparable stability in pertrochanteric fracture fixation within the TFNa system. Femoral neck component choice does not significantly affect mechanical complication rates or clinical outcomes. Rather, optimal implant placement and a postoperative TAD under 20 mm are key to successful outcomes. These results support prioritizing surgical precision over component selection in managing pertrochanteric fractures.

**Level of Evidence:**

Level III. See Instructions for Authors for a complete description of levels of evidence.

## Background

Trochanteric (pertrochanteric and intertrochanteric) fractures of the proximal femur are among the most common and consequential fractures occurring in the older adult population, typically in patients aged older than 60 years^1^. Patients who experience a trochanteric fracture have 1-year mortality rates ranging from 20% to 30%^2-5^. The majority of these fractures are treated surgically through fracture reduction and stabilization. While extramedullary fixation has long been the traditional treatment method for trochanteric fractures, the advancement of cephalomedullary systems, such as the TFNa (trochanteric femoral nail advanced, DePuy Synthes), has increased in popularity^6,7^ because of a shorter operative time, less intraoperative blood loss^8^, a shorter postoperative length of stay^9^, and improved radiographic outcomes^10^. Nevertheless, 7% to 20% of patients who survive the first postoperative year require reoperation because of mechanical complications^11-13^. Cephalomedullary nails, such as the TFNa, are designed to stabilize fractures by being inserted into the medullary canal of the femur, providing internal support to the bone while allowing controlled compression at the fracture site and preventing rotation. The surgeon has the option to choose between two different femoral neck components, a predrilled lag screw or a helical blade. However, there remains debate regarding which option provides optimal fixation^14-16^.

Unlike the lag screw, where bone is lost by drilling before insertion, the helical blade compacts the surrounding cancellous bone to improve femoral head stability^17^. The helical blade was introduced with the goal of enhancing fixation strength, particularly in osteoporotic bone, and thereby reduces the risk of cutout. While biomechanical investigations tend to favor the helical blade, citing its ability to minimize bone loss and provide enhanced holding force owing to its direct fixation into the femoral head^18-20^, concerns have arisen regarding complications associated with its use, especially medial cut-through, which is defined as medial migration of the helical blade with penetration into the acetabulum^21-24^.

The aim of this study was to evaluate and compare the performance of helical blades and lag screws for femoral neck fixation within the TFNa cephalomedullary nailing system and assess their ability to maintain fracture reduction over time through radiological and clinical outcomes. By providing a comprehensive assessment, this study seeks to generate actionable insights and recommendations to support clinical decision making in the management of trochanteric fractures.

## Methods

This study was designed as a prospective cohort study at a single institution with 12 board-certified orthopaedic surgeons, aiming to evaluate the impact of femoral neck component choice—helical blades versus predrilled lag screws—on radiological and clinical outcomes in AO/OTA 31A1-31A2 (pertrochanteric) and 31A3 (intertrochanteric) fractures treated with the short TFNa. Patients aged 50 years or older were consecutively allocated to either the screw or the blade cohort until each cohort reached 100 patients with a minimum follow-up of 3 months by mid-2024. Ethics approval was obtained before study commencement.

A flowchart of the patient inclusionII process in the screw and blade cohorts is included in Appendix 1.

The primary outcome was the maintenance of postoperative reduction at the 3-month follow-up. Radiographic parameters included the following:

1. Change in the central placement of the femoral neck screw as measured by the tip-apex distance (TAD) compared with the immediate postoperative value
2. Shortening of the femoral neck as measured by the sliding of the femoral neck component inside the nail. To account for femoral rotation, corrections were applied using the ratio of the radiographic screw or blade length to its actual length, as described by Tsukada et al.^25^
3. Change in the NSA in the anteroposterior view compared with the immediate postoperative value. A corrected NSA was calculated using the known implant angle, following the method described by Parry et al., to account for variations in leg rotation between radiographs^26^

An additional subgroup analysis of the primary outcome was performed for pertrochanteric and intertrochanteric fractures.

Secondary outcomes were as follows:

1. Occurrence and reasons for mechanical complications within a follow-up period of 1 year
2. Patient-reported outcome measures (PROMs), including EQ-5D-5L and EQ VAS scores
3. Number of patients deceased after 12 months

Surgical technique was standardized. Implant diameter (10, 11, or 12 mm) and nail angle (125° or 130°) were selected based on the contralateral femoral anatomy as assessed on an anteroposterior radiograph. When a lag screw was used, the femoral head and neck were predrilled with a cannulated tapered drill. The femoral neck component was secured against rotation using the proximal set screw. Controlled sliding was enabled by turning the set screw counterclockwise by half a turn once the implant was fully seated. All implants were distally locked in a static position. Immediate full weight-bearing was permitted for all patients postoperatively.

Data were prospectively collected from the hospital's electronic medical records. Baseline parameters included age, sex, American Society of Anesthesiologists (ASA) classification, side of injury, and fracture classification. Baseline radiographic data included the Singh Index for Osteoporosis^27^, the Baumgaertner Reduction Quality Criteria (BRQC)^28,29^, the Chang Reduction Quality Criteria (CRQC)^30^, the TAD^28^, restoration of NSA (absolute difference between NSA of the operated hip and contralateral healthy side), and screw/blade positioning according to the Cleveland zones^31^. Each radiographic measurement was assessed by two experienced surgeons (M.W. and J.H.) on the basis of a predetermined method. PROMs were collected at 6-week and 3-month follow-up appointments.

We used descriptive analysis to summarize the characteristics of the study population. Continuous variables were compared using Student *t*-test and categorical data with Fisher exact test. Statistical significance was set at p < 0.05. Sample size was based on detecting a 2-mm difference in TAD, using data from Yam et al., who reported a mean TAD of 19 (±5) mm^32^. With 100 patients per group, the study had 80% power (α = 0.05). Analyses were performed using Jamovi v2.3.3.0.

## Results

### Baseline Data

The study included 201 patients, including 101 patients in the blade cohort and 100 in the screw cohort. Table I presents the demographic data of the 2 cohorts. Baseline characteristics were similar in both cohorts.

**TABLE I:** Demographic and Clinical Data

Characteristic	Blade Cohort	Screw Cohort	p
No. of patients	101	100	
Mean age, years (range)	79.4 (47-99)	79.5 (47-99)	0.957*
Female sex, n (%)	77 (76.2)	70 (70.0)	0.343†
Left-sided hip fracture, n (%)	62 (61.4)	60 (60.0)	0.886†
ASA score, n (%)			1.0†
I	4 (4.0)	3 (3.0)	
II	29 (28.7)	30 (30.0)	
III	60 (59.4)	59 (59.0)	
IV	8 (7.9)	8 (8.0)	
Contralateral side			
DHS (%)	2 (2.0)	1 (1.0)	
Nail, n (%)	3 (3.0)	3 (3.0)	
THA, n (%)	2 (2.0)	5 (5.0)	
Hemiarthroplasty, n (%)	1 (1.0)	1 (1.0)	
Unoperated, n (%)	93 (92.0)	90 (90.0)	
SIO, mean (±SD)	2.62 (±0.7)	2.76 (±0.7)	0.170*
I, n (%)	6 (5.9)	4 (4.0)	
II, n (%)	33 (32.7)	29 (29.0)	
III, n (%)	55 (54.5)	54 (54.0)	
IV, n (%)	7 (6.9)	13 (13.0)	
V, n (%)	0	0	
VI, n (%)	0	0	
AO/OTA fracture type			0.365†
Pertrochanteric (%)	96 (95.1)	92 (92.0)	
AO31A1, n (%)	41 (40.6)	32 (32.0)	
AO31A2, n (%)	55 (54.5)	60 (60.0)	
Intertrochanteric			
AO31A3, n (%)	5 (4.9)	8 (8.0)	
Implant specification			
Nail thickness (%)			0.106†
10 mm	61 (59.4)	54 (54.0)	
11 mm	32 (31.7)	28 (28.0)	
12 mm	8 (7.9)	18 (18.0)	
Blade/Screw length in mm, mean (±SD)	95.6 (±8.5)	97.0 (±8.3)	0.236*
Nail angle (%)			0.238†
125°	70 (69.3)	61 (61.0)	
130°	31 (30.7)	39 (39.0)	

ASA = American Society of Anesthesiologists, AO = Arbeitsgemeinschaft für Osteosynthesefragen, FU = follow-up, OTA = Orthopaedic Trauma Association, SIO = Singh Index for osteoporosis, SD = standard deviation, and THA = total hip arthroplasty.

* Student's *t*-test.

† Fisher's exact test.

The quality of reduction assessed immediately postoperatively was demonstrated to be superior in the blade cohort according to both the BRQC and CRQC. In the screw cohort, the TAD was 1.5 mm lower than that in the blade cohort. The restoration of NSA was similar in both cohorts (Table II).

**TABLE II:** Baseline Postoperative Radiographic Reduction and Implant Position Measurements

Measurement	Blade Cohort n = 101	Screw Cohort n = 100	Difference Δ (95% CI)	Effect size	p
BRQC					0.001*
Poor, n (%)	10 (9.9)	26 (26.0)	−16.1		
Acceptable, n (%)	51 (50.5)	53 (53.0)	−2.5		
Good, n (%)	40 (39.6)	21 (21.0)	18.6		
CRQC					<0.001*
Poor, n (%)	0	1 (1.0)	−1.0		
Acceptable, n (%)	37 (36.6)	67 (67.0)	−30.4		
Excellent, n (%)	64 (63.4)	32 (32.0)	31.4		
TAD in mm, mean (±SD)	16.8 (±4.67)	15.3 (±4.18)	1.49 (0.26-2.73)	0.34	0.018†
TAD>20 mm, n (%)	25 (24.8)	15 (15.0)	+9.8		0.111*
Calcar displacement					
Displaced calcar lateral view, n (%)	21 (20.8)	21 (21.0)	−0.2		1.0*
Anterior displacement +, n (%)	5 (5.0)	14 (14.0)	−9.0		
Posterior displacement −, n (%)	16 (15.8)	7 (7.0)	8.8		
Displaced calcar AP view, n (%)	41 (40.6)	30 (30.0)	10.6		0.140*
Medial displacement +, n (%)	37 (36.6)	27 (27.0)	9.6		
Lateral displacement −, n (%)	4 (4.0)	3 (3.0)	1.0		
Restoration of NSA, absolute side difference in °, mean (±SD)	6.8 (±5.58)	7.16 (±6.0)	−0.36 (−1.99-1.27)	−0.06	0.662†

AP = anteroposterior, BRQC = Baumgaertner reduction quality criteria, CI = confidence interval, CRQC = Chang reduction quality criteria, NSA = neck-shaft angle, SD = standard deviation, and TAD = tip-apex distance.

* Fisher's exact test.

† Student's *t*-test.

The femoral neck component was centrally positioned in the majority of patients in both the blade (55.4%) and screw cohorts (61.0%). A figure illustrating the femoral neck component placement according to the Cleveland zones is provided in Appendix 2.

Table III presents the overall differences in the three primary outcomes assessing the maintenance of postoperative reduction between the two cohorts, as well as the subgroup analyses for pertrochanteric and intertrochanteric fractures.

**TABLE III:** Results of Primary Outcome Measures Divided by Fracture Type

Measurement	Blade Cohort	Screw Cohort	Difference Δ (95% CI)	Effect Size	p
Overall	n = 101	n = 100			
Change of TAD in mm, mean (±SD)	2.66 (±2.18)	2.43 (±4.87)	0.23 (−0.82 to 1.28)	0.06	0.667*
Femoral neck shortening, mm, mean (±SD)	6.25 (±4.58)	5.84 (±7.66)	0.42 (−1.34 to 2.17)	0.07	0.642*
Change of NSA, absolute value°, mean (±SD)	3.57 (±3.07)	4.16 (±4.20)	-0.59 (−1.61 to 0.44)	−0.16	0.261*
Pertrochanteric (AO31A1+2)	n = 96	n = 92			
Change of TAD in mm, mean (±SD)	2.61 (±2.04)	2.51 (±5.06)	0.10 (−1.00 to 1.20)	0.03	0.856*
Femoral neck shortening, mm, mean (±SD)	6.10 (±4.63)	5.87 (±7.94)	0.23 (−1.63 to 2.09)	0.04	0.807*
Change of NSA, absolute value°, mean (±SD)	3.58 (±3.12)	4.21 (±4.30)	−0.85 (−2.37 to 0.67)	-0.17	0.253*
Intertrochanteric (AO31A3)	n = 5	n = 8			
Change of TAD in mm, mean (±SD)	3.68 (±4.30)	1.58 (±1.30)	2.10 (−1.41 to 5.61)	0.75	0.214*
Femoral neck shortening, mm, mean (±SD)	9.05 (±2.38)	5.40 (±3.02)	3.65 (0.133 to 7.16)	1.30	0.043*
Change of NSA, absolute value°, mean (±SD)	3.31 (±1.91)	3.52 (±2.96)	−0.22 (−3.52 to 3.08)	-0.08	0.888*

CI = confidence interval, AP = anteroposterior, NSA = neck-shaft angle, SD = standard deviation, and TAD = tip-apex distance.

* Student *t*-test.

Appendix 3 presents the results of the post hoc power analysis, which demonstrated adequate power for the pertrochanteric subgroup but insufficient power for the intertrochanteric subgroup due to the small sample size.

Table IV summarizes the results of all secondary outcome measures. Mechanical complications occurred more frequently in the screw cohort than in the blade cohort (6.0% vs. 4.0%), though this difference was not statistically significant. Similarly, no significant differences in follow-up PROMs were observed between cohorts. Both groups showed gradual improvement in PROMs from 6 weeks to 3 months postoperatively.

**TABLE IV:** Results of Secondary Outcome Measures

	Blade cohort n = 101	Screw cohort n = 100	p
Mechanical complications total, n (%)	4 (4.0)	6 (6.0)	0.537*
Nail cutout, n (%)	3 (3.0)	3 (3.0)	
Implant failure, n (%)	0	1 (1.0)	
Irritation by laterally protruded blade/screw, n (%)	0	2 (2.0)	
LLD and loss of offset, n (%)	1 (1.0)	0	
Management of complication			
Conversion to THA, n (%)	2 (2.0)	3 (3.0)	
Conversion to Hemiarthroplasty, n (%)	0	1 (1.0)	
Screw exchange, n (%)	0	2 (1.0)	
Nonoperative, n (%)	2 (2.0)	0	
PROMS			
Six-week follow-up			
EQ5D score, mean (±SD)	11.2 (±4.73)	11.8 (±4.4)	0.360†
EQ-VAS score, mean (±SD)	64.8 (±17.9)	66.0 (±20.1)	0.684†
Three-month follow-up			
EQ5D score, mean (±SD)	10.9 (±3.47)	10.6 (±4.14)	0.611†
EQ-VAS score, mean (±SD)	71.4 (±18.1)	71.3 (±18.2)	0.980†
Patients deceased at 1-year FU, n	6	6	1.0*

EQ5D = EuroQol-5 dimension, EQ-VAS = EuroQol Visual Analog Scale, FU = follow-up, LDD = leg length discrepancy, PROMS = patient-reported outcomes measures, SD = standard deviation, and THA, total hip arthroplasty.

* Fisher exact test.

† Student *t*-test.

Table V provides an overview of the odds ratios for the risk factors associated with mechanical complications.

**TABLE V:** ORs for the Risk Factors Associated with Mechanical Complications

	Mechanical Complications	
Variable	OR (95% CI)	p
Female Sex	0.85 (0.21-3.41)	0.730
Use of Blade	0.646 (0.18-2.36)	0.537
Use of 130° nail	0.515 (0.14-1.85)	0.322
Left-sided injury	1.54 (0.39-6.15)	0.743
PO Calcar displacement AP	1.89 (0.53-6.78)	0.328
PO Calcar displacement Lateral	1.67 (0.41-6.76)	0.439
PO TAD>20 mm	4.4 (1.22-16.2)	0.023

AP = anteroposterior, OR = odds ratio, PO = postoperative, and TAD = tip-apex distance.

## Discussion

The most significant finding of this study is that helical blades and lag screws demonstrated comparable ability to maintain fracture reduction over time in pertrochanteric fracture fixation when used within a single cephalomedullary nailing system, the short TFNa. The choice of femoral neck component was not identified as a risk factor of mechanical complications. However, our findings support the association between a TAD greater than 20 mm and an increased risk of mechanical complications.

Pertrochanteric fractures exhibit different stability and biomechanical behavior compared with intertrochanteric fracture patterns^33^ and were therefore analyzed separately. Femoral neck shortening, changes in NSA, and changes in TAD were similar between the cohorts among pertrochanteric fractures, indicating that both blades and screws demonstrated adequate positional stability within the femoral head. The current literature focuses on cutout as the primary end point, representing the final consequence of fixation failure; however, no study has specifically evaluated the ability of screws or blades to maintain fracture reduction during the postoperative period. Cases involving loss of reduction without progressing to cutout have not been investigated, despite evidence suggesting that patients with better reduction quality tend to achieve more favorable clinical outcomes^34^. Although computational modeling has suggested that the helical blade enhances femoral head stability by compacting the bone around the implant, whereas the screw may lead to bone loss due to predrilling^17^, our clinical findings do not support this theoretical advantage. Among intertrochanteric fractures, femoral neck shortening was greater in the blade cohort than in the screw cohort. However, the small sample size in this subgroup precludes any definitive association with the choice of femoral neck component.

Our results demonstrated that the choice of femoral neck fixation component was not associated with mechanical complications (blade use, odds ratio [OR] = 0.646 (95% confidence interval [CI] = 0.18-2.36), p = 0.537). Published clinical studies comparing blades and screws are limited by small sample sizes^23^ and the fact that they compare these components across different implant manufacturers rather than within a single implant system^16,35-37^. This heterogeneity in study design and implant types contributes to conflicting results in recent meta-analyses. For example, a systematic review by Ng et al. suggested that the femoral neck component is not a risk factor of implant cutout, emphasizing instead the importance of factors such as central screw placement in predicting cut-out risk^38^. By contrast, a meta-analysis by Kim et al. highlighted a higher fixation failure rate associated with helical blades^39^. Furthermore, the few clinical studies in the literature comparing screws versus blades within the same implant system^23,40^ do not sufficiently assess possible risk factors contributing to fixation failure, including the quality of fracture reduction^41^, NSA malreduction exceeding 5° of varus or 15° of valgus^42^, a TAD surpassing 25 mm^28^, improper femoral neck screw placement^43^, and the direction of calcar displacement^25,30^.

Our results revealed a statistically significant association between a TAD greater than 20 mm and an increased risk of mechanical complications (OR = 4.4 (95% CI = 1.22-16.2), p = 0.023). Shorter TAD values are considered mechanically advantageous. While earlier studies on extramedullary implants such as the dynamic hip screw identified a TAD greater than 25 mm as a risk factor of screw cutout^28,43^, more recent evidence suggests that modern cephalomedullary systems may require stricter thresholds, with several studies reporting an increased risk of cutout when the TAD exceeds 20 mm^44-47^.

The mean postoperative TAD values observed in both cohorts are consistent with those reported in previous studies^48,49^ but were significantly lower in the screw cohort than in the blade cohort (15.3 vs. 16.8 mm, p = 0.018); however, the difference was small and unlikely to be clinically relevant.

Postoperative reduction quality and implant positioning differed between cohorts. The screw group had 16.1% more “poor” and 18% fewer “good” reductions according to the BRQC. Superior placement of the femoral neck component, associated with poor prognosis, occurred more often with screws (8 vs. 4 patients), while inferior placement, associated with better outcomes, was more frequent with blades (26.7% vs. 16.0%). Although not statistically significant, these factors may explain the slightly higher rate of mechanical complications in the screw group (6.0% vs. 4.0%). The quality of fracture reduction is a surgeon-dependent factor^5,50^ and is achieved before the insertion of the cephalomedullary and femoral neck components. However, the extent to which the choice of femoral neck component influences the risk of reduction loss during insertion could not be assessed in this study, as intraoperative radiographs of fracture reduction before implant placement were not available.

Six patients in each cohort died within 1 year, though these numbers do not represent true 1-year mortality rates, as only patients with at least 3 months of follow-up were included. Further limitations of this study include the absence of standardized patient randomization, leading to potential selection bias and confounding variables that may have influenced treatment allocation and outcomes. However, key confounding variables, such as age, sex distribution, and osteoporosis grade, were evenly distributed between cohorts. The relatively short follow-up period of 3 months is another limitation, as it may not fully capture the complete recovery trajectory following trochanteric hip fracture surgery.

## Conclusion

This study revealed no significant difference between helical blades and lag screws in maintaining fracture reduction with the short TFNa for pertrochanteric fractures. In this subgroup, both components showed similar positional stability, with comparable femoral neck shortening, NSA, and TAD changes. Owing to insufficient statistical power, no definitive conclusions can be drawn for intertrochanteric fractures. The choice of component was not associated with mechanical complications; only a postoperative TAD >20 mm was a significant risk factor. These results suggest that proper implant placement and reduction quality are more important than implant type in preventing mechanical failure, and that the helical blade offers no clear advantage over the lag screw in the treatment of pertrochanteric fractures.

## Appendix

Supporting material provided by the authors is posted with the online version of this article as a data supplement at jbjs.org (http://links.lww.com/JBJSOA/A967). This content was not copyedited or verified by JBJS.
